# Detection and Identification of Hematologic Malignancies and Solid Tumors by an Electrochemical Technique

**DOI:** 10.1371/journal.pone.0153821

**Published:** 2016-04-26

**Authors:** Yujie Wang, Bowen Zhang, Xiaoping Zhang, Xuemei Wang, Jian Cheng, Baoan Chen

**Affiliations:** 1 Department of hematology and oncology (key Discipline of Jiangshu Province), Zhongda Hospital, School of Medicine, Southeast University, Nangjing, Jiangsu Province, People’s Republic of China; 2 State Key Laboratory of Bioelectronics (Chien-Shiung Wu Laboratory), Southeast University, Nanjing, Jiangsu Province, People’s Republic of China; Queen's University Belfast, UNITED KINGDOM

## Abstract

**Purpose:**

Develop and evaluate an electrochemical method to identify healthy individuals, malignant hematopathic patients and solid tumor patients by detecting the leukocytes in whole-blood.

**Methods:**

A total of 114 individual blood samples obtained from our affiliated hospital in China (June 2015- August 2015) were divided into three groups: healthy individuals (n = 35), hematologic malignancies (n = 41) and solid tumors (n = 38). An electrochemical workstation system was used to measure differential pulse voltammetry due to the different electrochemical behaviors of leukocytes in blood samples. Then, one-way analysis of variance (ANOVA) was applied to analyze the scanning curves and to compare the peak potential and peak current.

**Results:**

The scanning curve demonstrated the specific electrochemical behaviors of the blank potassium ferricyanide solution and that mixed with blood samples in different groups. Significant differences in mean peak potentials of mixture and shifts (ΔEp (mV)) were observed of the three groups (P< = 0.001). 106.00±9.00 and 3.14±7.48 for Group healthy individuals, 120.90±11.18 and 18.10±8.81 for Group hematologic malignancies, 136.84±11.53 and 32.89±10.50 for Group solid tumors, respectively. In contrast, there were no significant differences in the peak currents and shifts.

**Conclusions:**

The newly developed method to apply the electrochemical workstation system to identify hematologic malignancies and solid tumors with good sensitivity and specificity might be effective, suggesting a potential utility in clinical application.

## Introduction

Hematologic malignancies have become a big threat to individuals of all ages and affected the living quality of people worldwide [1, 2]. Although latest and suitable chemotherapy drugs on targeted tumors are important to improve the clinical outcomes of patients with hematologic malignancies, accurate early detection should be prioritized. Current methods and techniques in the diagnosis of hematologic malignancies include biopsy [3], peripheral blood testing [4], bone marrow biopsy [5], immunology testing [6], flow cytometry [7], radiologic examination[8], chromosome analysis and DNA sequencing technology [9], *etc*. Due to the disadvantages such as high cost, time consuming, complexity, and radioactive pollution of above mentioned clinical detection techniques [10], it is interest to develop a new user-friendly and cost-effective technology with good sensitivity to detect hematologic malignancies. Recently, the superior advantages of bio-electrochemistry applied in clinical diagnostics such as high sensitivity, portability and rapid detection have attracted attention of manyresearchers and physicians [11–14].

Electrochemical methods have been widely used in molecular biology [15, 16] and cytobiology [17, 18]. For example, using differential pulse voltammetry (DPV) to detect the leukemia cells with a limit of 1.0×10^4^ cell/mL by methoxysilyl-butyrylchitosan/Au nanoparticles (NPs) was reported by Du and colleagues [19]. Similar result was found by He *et al*. [20] with the leukemia detection limit of 1.0×10^3^ cell/mL after incubation time of 120 min using Au NPs in DPV. Moreover, Zhu and coworkers [21] developed a novel electrochemical platform based on aptamer–cell–aptamer sandwich architecture for the detection of Michigan cancer foundation-7 human breast cancer cells with high selectivity and sensitivity. Physiological activities mainly happen with directional transfer of electric charge [22] simultaneously from cells both in excitement and quiescent stage [23]. The biochemical reaction of cells, which is just like the electrochemical reaction in electrodes, is the foundation for basic physiology. In recent years, accumulating researches have demonstrated the feasibility of detection and identification of cells by electrochemical methods to distinguish normal and cancer cells[24, 25], as well as to identify drug-resistant or sensitive cells [26, 27] according to the particular responses in the electrode. However, there were few reports focusing on the application of electrochemical methods in the analysis of human blood. Several recent studies have identified the characteristics of different cells [28, 29] or drugs [30, 31] via detecting impedance of cells. The aim of this study was to develop and evaluate a method to identify hematologic malignancies and solid tumors by detecting the electrochemical characteristics of leukocytes in the peripheral blood of patients, which might be effectively used in clinical application.

## Materials and Methods

### Ethics Statement

Human whole-blood samples were obtained from normal persons and patients at the department of clinical laboratory in Zhongda Hospital, Affiliated to Southeast University in accordance with the standard operating protocols of the National Guide to Clinical Laboratory Procedures (Third Edition, 2006) that were approved by the Department of Medical Administration, Ministry of Health, People’s Republic of China. Our study was included in the Key Medical Projects of Jiangsu Province (BL2014078) which had been reviewed and approved by the local ethics committee of Zhongda Hospital, Affiliated to Southeast University (2015ZDSYLL024.1). All procedures used in this study were adhered to Declaration of Helsinki Ethics, and verbal informed consent was obtained from all participants before enrollment.

Because it only required a small amount blood of each samples for the experiment (about 0.5 mL), the experimental subjects were the residual peripheral blood samples derived from laboratory department and had been applied for medical testing. We didn’t need to take additional information and blood samples from them. In this study, the obtained electrochemical test data were going to be analyzed anonymously, such as ages and gender. Moreover, the patients’ health, safety and privacy were not affected, we could be ensured that the obtained data were only used for this research, the information were confidential and protected as well. Therefore, the ethic committee of Zhongda Hospital, Affiliated to Southeast University specifically decided that we didn’t need the written informed consent, and the verbal informed consent was obtained from all study participants after the nature of the study was explained by senior physicians who were in our study, and documented by the researchers in our study.

### Patient Population

From June to August 2015, 114 whole-blood samples were obtained randomly from the department of clinical laboratory in Zhongda Hospital, Affiliated to Southeast University. We divided them into three groups according to their different physical condition. There were 35 people in Group healthy individuals, 41 people in Group hematologic malignancies and 38 people in Group solid tumors. The data were extracted from the electronic medical records of Zhongda Hospital, such as genders and ages. The demographic statistics and clinical characteristics are shown in Table 1. The patients with malignant hematopathic were diagnosed by senior physicians referring to the bone marrow cell morphology and pathology, and the patients with solid tumor were diagnosed via biopsy.

**Table 1:** The demographic statistics and clinical characteristics of patients.

Group	Age (years)	Male/Female
**Healthy individuals**	46.00±5.63	16/19
**Hematologic malignancies**	48.24±18.72	21/20
**Solid tumors**	52.58±13.12	18/20
**P-value**	0.884	0.123

### Instruments and Reagents

**Fig 1:**
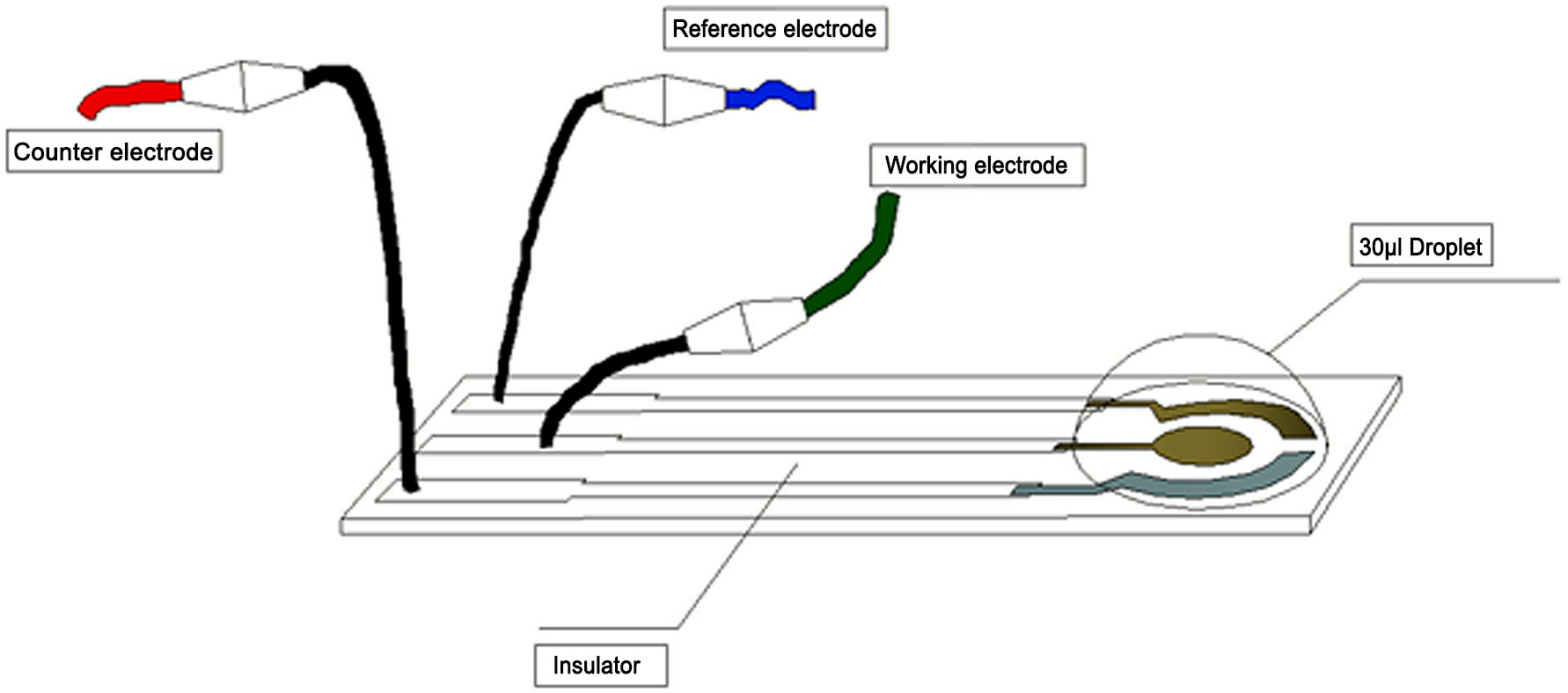
A screen-printed three-electrode system.

Whole-blood samples were performed on a EmStat Electrochemical Workstation (EmStat in PalmSens BV, Netherlands) at room temperature [32].The three-electrode system, screen-printed carbon electrode (SPCE) were provided by State Key Laboratory of Bioelectronics (Chien-Shiung Wu Laboratory), Southeast University and used in all electrochemical measurements (Fig 1). Experiments were carried out in the potassium ferricyanide solution with the three-electrode system, which was connected with the electrochemical workstation to measure differential pulse (DPV). The measurement parameters for DPV were chosen as follows, scanning voltammetry from -0.2 V to 0.4 V, scanning rate 25 mV/s and pulse period 0.2 s.

The ammonium chloride aqueous solution was prepared by dissolving 0.83 g NH_4_Cl in 100 mL deionized water to dissociate erythrocytes, the phosphate buffer solution (PBS, 0.01 mM, pH = 7.4) was used as a medium, and the potassium ferricyanide solution (0.01 mM) was used as redox probe. All the experimental water was ultrapure water and the experiments were carried out at ambient temperature.

### Processing and Detecting of Samples

The isolation of leukocytes was achieved by centrifugation the whole-blood sample in 0.83% NH_4_Cl solution at 1500 rpm for 15 min twice and removal of fragmentized erythrocyte. After that, the isolated leukocytes were washed once by PBS once and then suspended in PBS medium (300 μL). We extracted 50 μL leukocyte suspension and dripped it into 50 μL potassium ferricyanide solution.

Thereafter, we scan the 30 μL potassium ferricyanide solution (0.01 mM) on the surface of electrode via DPV as the standard comparison. After washing the electrode with the deionized water once and drying it, 30 μL potassium ferricyanide solution mixed with leukocyte suspension onto the surface of dry electrode was scanned repeatedly and a stable scanning curve was obtained.

### Statistical Analysis

Data were expressed as mean±standard deviation (SD) (normally distributed data) or as percentage frequencies. The comparisons were performed by one-way analysis of variance (ANOVA) at significance levels of p<0.05, and then the TurKey method was used for all pairwise comparisons after ANOVA tests. All the analysis were made using a standard statistical package (SPSS for Windows Version 18.0; Chicago, IL).

## Results

**Fig 2:**
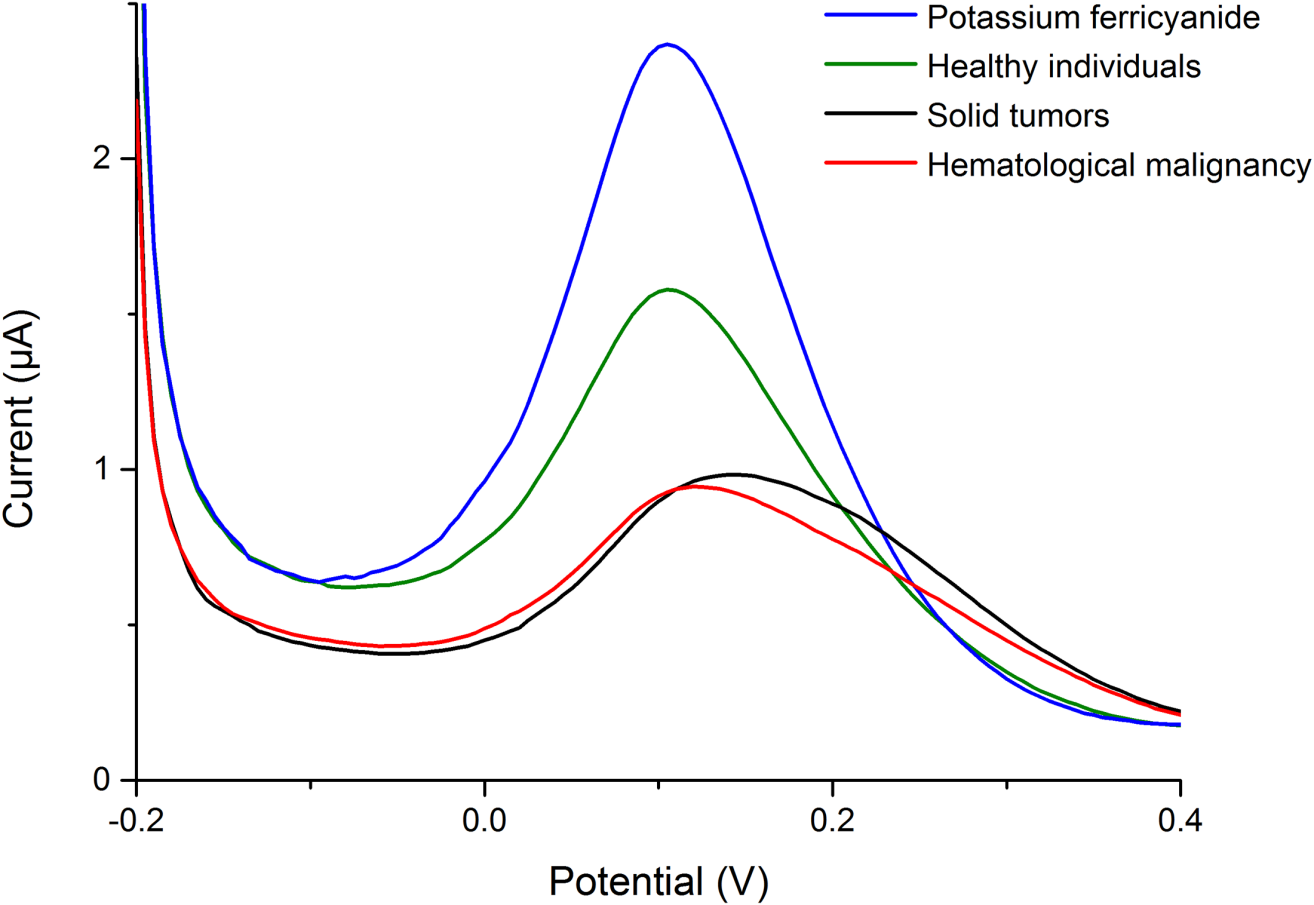
Typical curves of the potassium ferricyanide solution (0.01 mM) with/without leukocyte suspension scanned by the differential pulse voltammograms (DPV) techquine, scanning voltammetry from -0.2 V to 0.4 V and a 0.2 s pulse period.

As shown in Fig 2, the scanning curves represented the electrochemical behaviours of the blank potassium ferricyanide solution, as well as that mixed with leukocyte suspension. The peak potential, the peak current, and the calculated shifts were shown in Table 2. The mean peak potentials of potassium ferricyanide solution without leukocyte suspension were 102.86±8.07, 102.81±7.99, and 103.95±7.09 mV for Group healthy individuals, Group hematologic malignancies and Group solid tumors, respectively, while the numbers with leukocyte suspension were 106.00±9.00, 120.90±11.18, 136.84±11.53 mV for the three groups. The mean shifts of peak potential (ΔEp (mV)) were 3.14±7.48 mV in Group healthy individual, 18.10±8.81 mV in Group hematologic malignancies and 32.89±10.50 mV in Group solid tumor, with significant difference among them (P< = 0.001). The median peak potentials of potassium ferricyanide solution with/without leukocyte suspension, and the median shifts of peak potential (ΔEp (mV)) have presented in Table 3. All the pairwise comparisons were also significantly different when the TurKey method was applied. However, the mean shifts peak currents of potassium ferricyanide and the mixture were of no significant difference among the three groups (P = 0.470, 0.068, 0.257).

**Table 2:** The characteristics of the scanning curves. The standard deviations in electrochemical behavior of different samples.

Groups	Peak potential (mV)		Shifts of peak potential (mV)	Peak current (μA)		Shifts of peak current (μA)
	potassium ferricyanide	sample		potassium ferricyanide	sample	
**Healthy individuals**	102.86±8.07	106.00±9.00	3.14±7.48	1.28±0.51	0.85±0.41	0.43±0.54
**Hematologic malignancies**	102.81±7.99	120.90±11.18	18.10±8.81	1.18±0.58	0.68±0.26	0.50±0.49
**Solid tumors**	103.95±7.09	136.84±11.53	32.89±10.50	1.13±0.40	0.80±0.31	0.34±0.24
**P-value**	0.767	< = 0.001*	< = 0.001*	0.470	0.068	0.257

* Statistically significant value

**Table 3:** The characteristics of the scanning curves. The median in electrochemical behavior of different samples.

Groups	Peak potential (V)		Shifts of peak potential (V)	Peak current (μA)		Shifts of peak current (μA)
	potassium ferricyanide	sample		potassium ferricyanide	sample	
**Healthy individuals**	0.09999	0.105	0.005	1.198	0.705	0.37
**Hematologic malignancies**	0.09999	0.12	0.02	1.088	0.594	0.431
**Solid tumors**	0.108	0.135	0.0325	1.1315	0.7402	0.2705

**Fig 3:**
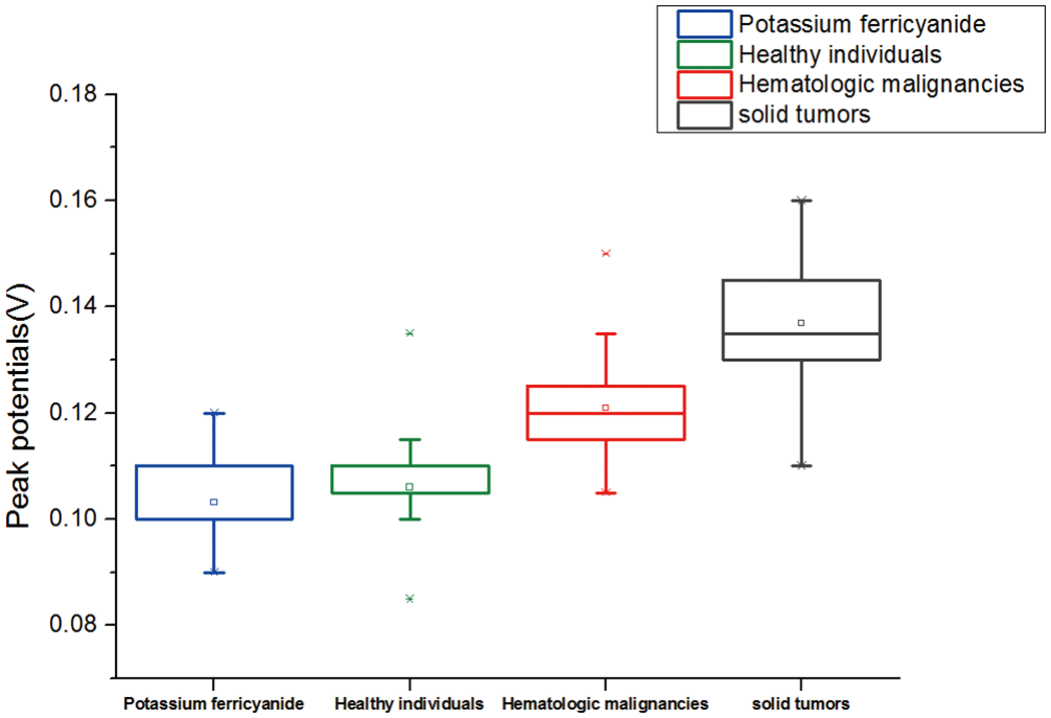
Box plots of peak potentials for blank potassium ferricyanide solution (0.01 mM) and that mixed with leukocyte suspension.

**Fig 4:**
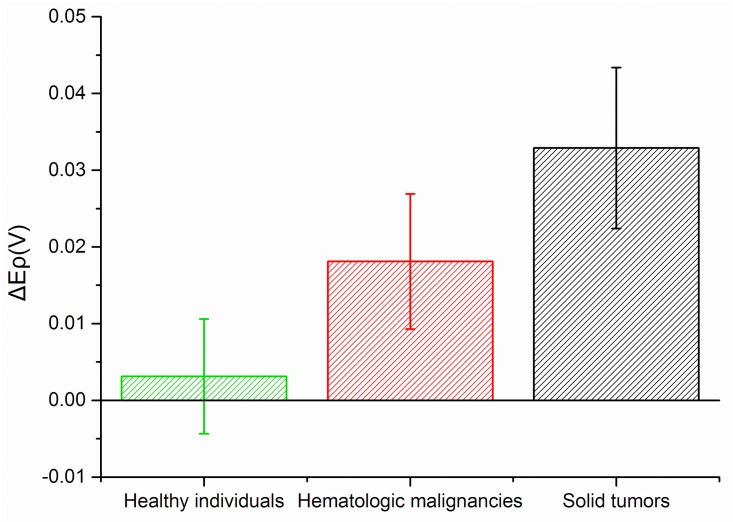
Box plots of potassium hexacyanoferrate solution (0.01 mM) mixed with samples from Group healthy individuals, Group hematologic malignancies and Group solid tumor.

As shown in Fig 3, there were slight differences in the relevant peak potentials of blank potassium ferricyanide solution among the three groups, but the situation was total different when that was mixed with leukocyte suspension. To be specific, the peak potential shifts showed gradual increase in the order of Group healthy individuals, Group hematologic malignancies and Group solid tumors. In addition, mean shifts of peak potential in three groups were also detected (Fig 4), which follows the order: Group healthy individuals < Group hematologic malignancies < Group solid tumors. The above results suggested that it is possible to utilize this technique to identify different types of blood samples through corresponding peak potential shifts. More broadly, it can be used to readily detect whether a person is either healthy or with hematologic malignancies or solid tumors.

## Discussion

Reports have shown that the redox system in living cells and the electrode may be connected by proteins across the membrane system of the cells [33–35]. Coenzyme A (CoA) existing in the cell wall played a similar role of mediating an electron transfer between the cells and the electrode [36]. Leukocytes in human blood have different metabolisms under different physiological and pathological conditions [37–40]. The abnormal metabolism of malignant cells and the heteromorphosis of plasma membrane would further change proteins and CoA, resulting in the abnormal electron transfer between the cells and the electrode.

It is well known that living organisms are complex electrochemical systems [41]. Redox centers in living cells would give response to the electrode when they were under the potential scan [42], however, it would not apply to tumor cells, for which are in hypoxic environment [43, 44]. [Fe(CN)_6_]_3_/[Fe(CN)_6_]_4_ was generally used as the redox probe to measuring the electron transfer resistivity of the electrode [45]. The redox reaction of potassium ferricyanide (III) was very reversible [46–48], while the electrode redox of leukocytes were totally irreversible processes [49]. Based on the above discussion, we chose a potassium ferricyanide solution as the standard comparison in this study. The redox reaction of the potassium ferricyanide solution mixed with leukocyte suspension (30 μL) that extracted from blood samples was different from that of blank potassium ferricyanide solution, which could further lead to the changes of the scanning curves (Fig 2). The results of our experiments showed that the mean shifts (ΔEp (mV)) of the peak potentials between the potassium ferricyanide solution and the mixture were 3.14±7.48, 18.10±8.81, 32.89±10.50 mV in Group physical examinees, Group hematologic malignancies and Group solid tumor respectively (Table 2). The significant difference was a result of the different electron transfer between the cells and the electrode, which might be due to the changes of proteins and CoA in the malignant cells [33–40].

When the mixture of and leukocyte suspension was placed onto the surface of electrode, the shifts of the peak potentials, along with current peaks were both lower than blank potassium ferricyanide solution. The influence of current peak was reported to be associated with the concentration of the solution rather than the types of the solute [50, 51]. Huan *et al*. reported the peak current of potassium ferricyanide scanned by differential pulse voltammetry (DPV) decreased linearly with increasing L-serine concentration from 10 to 100 mM [52]. Laputkova *et al*. reported both the oxidation and the reduction current peaks of potassium ferricyanide were decreasing at the presence of glucose in concentration range from 10 to 320 mM by means of cyclic voltammetry (CV) [53]. In conclusion, the concentration of cell affected the current peak, whereas, the type of the cell affected the peak potentials. In the extreme case that there were ultralow concentration of cancer cells existing, we should also detect them and distinguish the blood sample from healthy people. In our experiment, the mean shifts between the current peak of the blank potassium ferricyanide solution and that mixed with leukocyte suspension were 0.43±0.54, 0.50±0.49, and 0.34±0.24 μA in Group healthy individuals, Group hematologic malignancies and Group solid tumor respectively, which were no significant difference (P = 0.257) among them (Table 2). Therefore, we could infer that the current peak didn’t help in the identification of different blood samples, which was the purpose of our research.

There are still some limitations in our research. Firstly, the types of blood samples were not further discriminated, for which leukemia and lymphadenoma may present slight difference in electrochemical behaviours. Moreover, the patients were in the different stages of chemotherapy, which may also affect the electrochemical characteristics of the leukocytes. Finally, the number of patients was not enough and all patient data were from the same hospital, which had adverse impact on the results of our experiment. In future studies, increasing the number and collecting multi-center information of patients would improve the clinical application value of monitoring results.

## Conclusion

According to the significant difference among the scanning curves of healthy individuals and patients with either hematologic malignancies or solid tumors, the electrochemical detection method may be a selective and convenient technique for the identification of hematologic malignancies and solid tumors in clinical applications. However, improving the accuracy and stability for the detection of particular hematologic malignancies needs further research.

## Supporting Information

S1 Initial Data The data of samples are detected by DPV of electrochemical workstation. (XLS)
